# Effect of iron overload from multi walled carbon nanotubes on neutrophil-like differentiated HL-60 cells

**DOI:** 10.1038/s41598-019-38598-4

**Published:** 2019-02-18

**Authors:** Yosuke Tabei, Hiroko Fukui, Ayako Nishioka, Yuji Hagiwara, Kei Sato, Tadashi Yoneda, Tamami Koyama, Masanori Horie

**Affiliations:** 1 Health Research Institute, National Institute of Advanced Industrial Science and Technology (AIST), 2217-14 Hayashi-cho, Takamatsu, Kagawa 761-0395 Japan; 2 Safety Evaluation Center, Showa Denko K.K., 1-1-1 Ohnodai, Midori-ku, Chiba-shi, Chiba 267-0056 Japan; 3 Institute for Advanced and Core Technology, Showa Denko K.K., 1-1-1 Ohnodai, Midori-ku, Chiba-shi, Chiba 267-0056 Japan

## Abstract

Multi walled carbon nanotubes (MWCNTs) are one of the most intensively explored nanomaterials because of their unique physical and chemical properties. Due to the widespread use of MWCNTs, it is important to investigate their effects on human health. The precise mechanism of MWCNT toxicity has not been fully elucidated. The present study was designed to examine the mechanisms of MWCNT toxicity toward human promyelocytic leukemia HL-60 cells. First, we found that MWCNTs decreased the viability of neutrophil-like differentiated HL-60 cells but not undifferentiated HL-60 cells. Because neutrophil-like differentiated HL-60 cells exhibit enhanced phagocytic activity, the cytotoxicity of MWCNTs is dependent on the intracellularly localized MWCNTs. Next, we revealed that the cytotoxicity of MWCNTs is correlated with the intracellular accumulation of iron that is released from the engulfed MWCNTs in an acidic lysosomal environment. The intracellular accumulation of iron was repressed by treatment with cytochalasin D, a phagocytosis inhibitor. In addition, our results indicated that iron overload enhanced the release of interleukin-8 (IL-8), a chemokine that activates neutrophils, and subsequently elevated intracellular calcium concentration ([Ca^2+^]_i_). Finally, we found that the sustained [Ca^2+^]_i_ elevation resulted in the loss of mitochondrial membrane potential and the increase of caspase-3 activity, thereby inducing apoptotic cell death. These findings suggest that the iron overload caused by engulfed MWCNTs results in the increase of IL-8 production and the elevation of [Ca^2+^]_i_, thereby activating the mitochondria-mediated apoptotic pathway.

## Introduction

Since the discovery of carbon nanotubes (CNTs) in 1991 by Iijima^1^, CNTs have attracted immense attention in the scientific and technological community. Because of their unique mechanical, chemical, and electrical properties^2^, which include high electrical conductivity, flexibility, elasticity, and thermal conductivity, CNTs have been widely studied and applied in polymer composition, microelectronics, and sensors^3^. Several studies have focused on the clinical application of CNTs, including nanomedicine and drug delivery systems^4^. However, the increased production and use of CNTs have raised concerns about the safety of industrial workers exposed to particulate aerosols produced during the CNT manufacturing and handling process.

In general, CNTs are classified into two groups: single walled carbon nanotubes (SWCNTs), which are composed of a single cylindrical sheet of graphene, and multi walled carbon nanotubes (MWCNTs), which consist of several concentric, coaxial, rolled-up graphene sheets. Previously, it was reported that SWCNTs are more toxic than MWCNTs^5^. However, there is accumulating evidence suggesting that MWCNTs induce lung inflammation, fibrosis, and granuloma formation^6–11^. In addition, it was reported that MWCNTs induce malignant mesothelioma in p53+/− mice^12^ and Fischer-344 rats^13^. The carcinogenicity of MWCNTs was also reported in rats after intraperitoneal injection and in mice after inhalation exposure^14,15^. Based on the results of animal studies, the International Agency for Research of Cancer has classified Mitsui-7 MWCNT as class 2B, a possible human carcinogen^16^. Indeed, Mitsui-7 MWCNTs were recently shown to induce lung cancer in rats by inhalation^17^. However, the elucidation of the toxicity or carcinogenicity determinants of MWCNTs is still incomplete.

The cytotoxicity of CNTs is attributed to their physicochemical parameters, such as size, shape, purity, and surface properties^18,19^. For example, long MWCNTs cannot be fully engulfed by macrophages and lead to frustrated phagocytosis and chronic inflammation^20,21^. Yamashita *et al*. demonstrated that long MWCNTs caused significant DNA damage and increased total cell number in abdominal lavage fluid, whereas short MWCNTs had less effect^22^. Opposite results have been reported as well. Han *et al*. found that exposure of C6 rat glioma cells to short MWCNTs induced more apoptotic cells than exposure to long MWCNTs^23^. In addition, it has been reported that short MWCNTs induced more TNF-ɑ production than long MWCNTs in THP-1 macrophages and RAW 264.7 cells^24,25^. These contrasting results can be attributed to the conditions for *in vitro* analysis and the cell types used for assays^26,27^. Thus, although a lot of studies have been reported about MWCNT toxicity, thorough understanding of the physicochemical parameters of MWCNT-mediated toxicity remains lacking.

The purpose of this study was to elucidate the cytotoxic effects of MWCNTs and investigate some of the underlying mechanisms by evaluating the intracellular accumulation of ferrous iron following the intracellular uptake of MWCNTs. Several studies have demonstrated that contamination with transition metals is one of the most important contributors to CNT-mediated cytotoxicity. Typically, iron, nickel, and cobalt are used as catalysts in the synthesis of CNTs. Among these metal catalysts, iron is considered to be the cause of genotoxicity and cytotoxicity of CNTs^28^. Although the toxic effects of iron have been associated with increased oxidative stress^29^ and inflammatory response^30^, the precise mechanisms of iron-mediated MWCNT toxicity and the interactions between physiological systems are not well understood. Therefore, in the present study, we investigated MWCNT-induced cytotoxicity and its impact on the mitochondria-mediated apoptotic pathway in human promyelocytic leukemia HL-60 cells that differentiated into neutrophil-like cells. Our data indicated that iron overload caused by MWCNTs triggered the production of IL-8 and the increase of intracellular calcium levels, and these were followed by the activation of the mitochondria-mediated apoptotic pathway.

## Results and Discussion

### Effect of MWCNTs on viability of HL-60 cells

**Figure 1:**
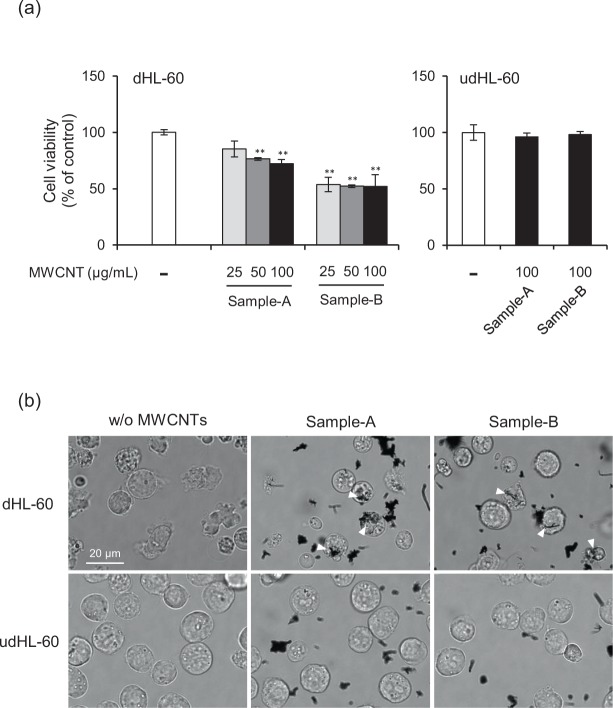
Effect of MWCNTs on cell viability. (**a**) dHL-60 and udHL-60 cells were exposed to MWCNTs for 24 h. Cell viability was measured using the WST-1 assay and the results are expressed as percentage of untreated controls. Values are means ± SD (n = 3). **P < 0.01 (versus untreated control, Dunnett, ANOVA). (**b**) Intracellular localization of MWCNTs. dHL-60 and udHL-60 cells were exposed to 25 μg/mL MWCNTs for 24 h, and then microscopic images were obtained. Engulfed MWCNTs were indicated by white arrowhead. Scale bar = 20 μm.

As a first experiment, we evaluated the cytotoxicity of two types of MWCNTs toward HL-60 that differentiated into neutrophil-like cells (hereafter referred to as dHL-60 cells) and undifferentiated cells (udHL-60 cells) using the WST-1 assay. According to the manufacturer’s data, sample-A has a tube diameter of 176 nm, a length of 5.2 μm, and a specific surface area of 14 m^2^/g, and sample-B (also known as Mitsui-7 or MWNT-7) has a tube diameter of 60 nm, a length of 10 μm, and a specific surface area of 25–30 m^2^/g. In order to establish the hydrodynamic diameter of the MWCNTs, we carried out the dynamic light scattering (DLS) measurement. Although DLS analysis mainly provides information of sphere shaped particles, the technique can provide hydrodynamic diameter information in the case of CNTs^31,32^. The results of DLS measurement indicated that the hydrodynamic diameter of MWCNTs increased in a time-dependent manner (Supplementary Fig. S1a,b) and slight sedimentation occurred in the culture medium. However, we did not observe any marked differences between sample-A and sample-B (Supplementary Fig. S1c,d). As shown in Fig. 1a, the viability of dHL-60 cells was decreased with increasing concentration of MWCNTs, and the cytotoxicity of sample-B was higher than that of sample-A. On the other hand, the cytotoxicity of MWCNTs toward udHL-60 cells was not observed (Fig. 1a, right panel). As dimethyl sulfoxide (DMSO)-differentiated HL-60 exhibited enhanced phagocytic activity^33^, our results suggest that the cytotoxicity of MWCNTs is dependent on the intracellularly localized MWCNTs. Indeed, microscopic images show that MWCNTs were incorporated into dHL-60 cells after incubation for 24 h, but not into udHL-60 cells (Fig. 1b).

### Iron release from MWCNTs and iron accumulation in HL-60 cells

**Figure 2:**
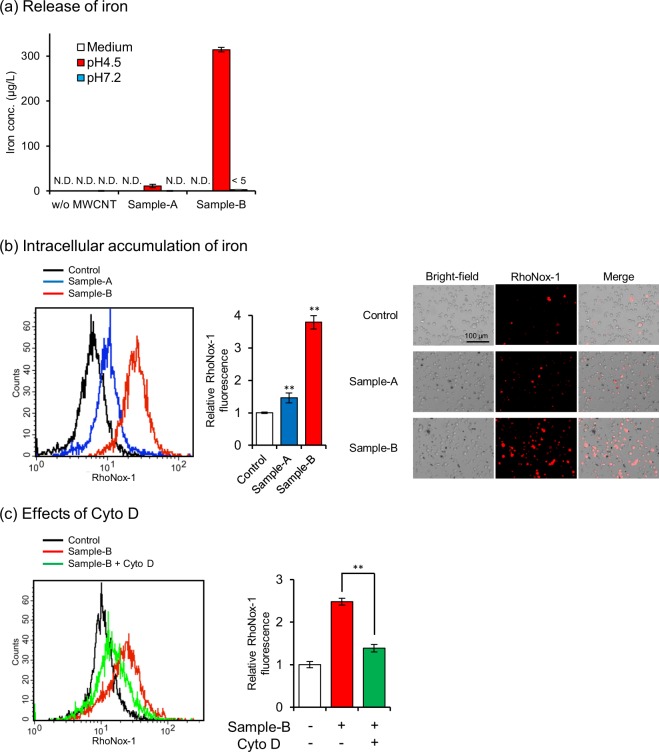
Iron release from MWCNTs. (**a**) Amounts of iron released from MWCNTs incubated at 37 °C for 24 h in culture medium, acidic condition (pH 4.5), or neutral condition (pH 7.2), as probed by ICP-MS. Acidic and neutral conditions were achieved by dispersing MWCNTs in citrate buffer and sodium phosphate buffer, respectively. (**b**) Intracellular accumulation of iron. dHL-60 cells were exposed to 100 μg/mL MWCNTs for 24 h and stained with RhoNox-1 for 1 h. Then, they were analyzed by flow cytometry and visualized under a fluorescence microscope. (**c**) Effects of Cyto D (0.5 μM) on the intracellular accumulation of iron in dHL-60 cells exposed to 100 μg/mL MWCNTs. Values are means ± SD (n = 3). **P < 0.01 (versus untreated control, Dunnett, ANOVA). Statistical comparisons between the two groups were carried out using the Student’s *t*-test.

Several studies have demonstrated that iron used as a catalyst in MWCNT synthesis plays a key role in MWCNT-induced cytotoxicity^34–36^. Then, we measured iron content in MWCNTs. X-ray fluorescence spectroscopy analyses demonstrated that iron contents of sample-A and sample-B were 9 ppm and 4206 ppm, respectively. In addition, using inductively coupled plasma-mass spectrometer (ICP-MS), we measured iron leakage from MWCNTs in culture medium and under two different conditions: one mimicking the lysosomal environment (pH 4.5, 37 °C) and the other, the cellular cytoplasmic environment (pH 7.2, 37 °C)^37–39^. As shown in Fig. 2a, we observed significant iron release from sample-B in the acidic condition and non-measurable release in the neutral condition and the culture medium. In contrast, sample-A did not release iron significantly under any conditions. Iron release from MWCNTs in the acidic condition was depended on their impurities, since sample-B contained large amounts of MWCNT-associated iron. Then, we assessed the intracellular accumulation of iron in dHL-60 cells exposed to MWCNTs using the RhoNox-1, which is a highly selective fluorescence probe for ferrous iron^40^. We observed a significant increase in intracellular iron levels in cells exposed to sample-B but not in cells exposed to sample-A, by flow cytometry and fluorescence microscopy (Fig. 2b). In addition, to investigate the role of the acidic environment of lysosomes in the release of iron, intracellular accumulation of iron was assessed in the presence of ammonium chloride, a lysosomotropic reagent that prevents lysosomal acidification^39,41^. As shown in Supplementary Fig. S2, the treatment with ammonium chloride attenuated the intracellular accumulation of iron. Previous studies have shown that the biodegradation of CNTs is mediated by peroxidase-driven processes^42^. Therefore, intracellular accumulation of iron is considered to be induced by biodegradation of MWCNTs on both lysosome- and peroxidase-mediated mechanisms. In addition, treatment of dHL-60 cells with cytochalasin D (Cyto D), a phagocytosis inhibitor, resulted in the attenuation of the increase of intracellular iron accumulation (Fig. 2c). These results indicated that iron was released from engulfed MWCNTs in the acidic lysosomal environment, and the accumulated ferrous iron induced MWCNT-mediated cytotoxicity toward dHL-60 cells. Hereafter, sample-A and sample-B are designated as _Low_Fe-CNT and _High_Fe-CNT, respectively, depending on their ability to release iron.

As ferrous iron initiates the Fenton reaction to generate hydroxyl radicals that induce oxidative stress, we measured acellular and intracellular ROS generation by MWCNTs. Indeed, it has been reported that MWCNT iron impurities to play a key role in inducing oxidative stress and reducing cell viability^34–36^. As shown in Supplementary Fig. S3a, the acellular ROS generation assay showed that MWCNTs slightly induced the oxidation of 2′,7′-dichlorodihydrofluorescin (DCFH) in a time-dependent manner. However, the oxidative potentials of _High_Fe-CNT were not different from those of _Low_Fe-CNT. Intracellular ROS generation is shown in Supplementary Fig. S3b. Exposing dHL-60 cells to MWCNTs resulted in slightly increased intracellular ROS levels. However, the increased intracellular ROS levels were not consistent with the accumulation of intracellular ferrous iron levels. Taken together, our results suggest that the cytotoxicity of MWCNTs toward dHL-60 cells is not only induced by oxidative stress mediated by the Fenton reaction.

### Effects of MWCNTs on release of interleukin-8

**Figure 3:**
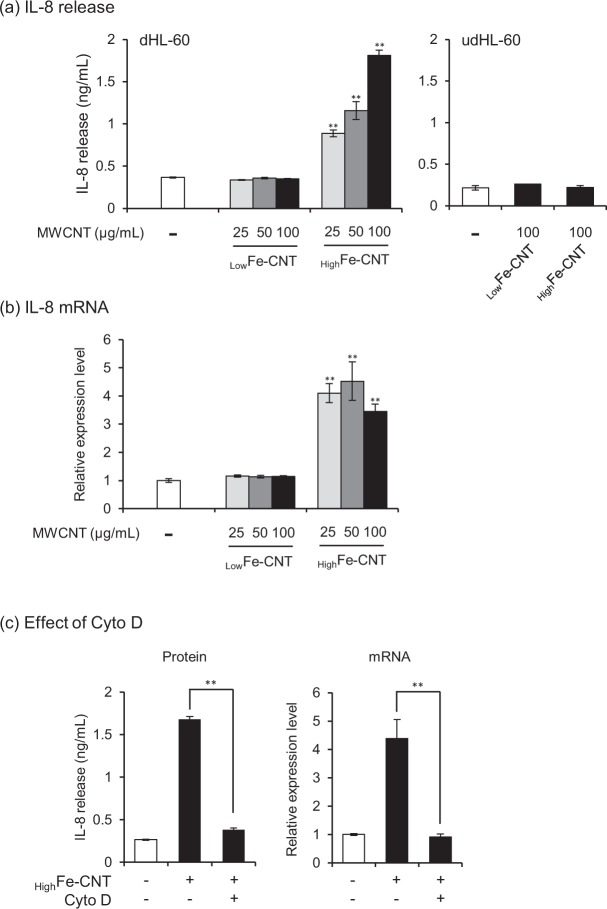
Effect of MWCNTs on IL-8 release and mRNA expression. (**a**) dHL-60 and udHL-60 cells were exposed to MWCNTs for 24 h and then IL-8 release into culture medium was determined using ELISA. (**b**) dHL-60 cells were exposed to MWCNTs for 24 h and then *IL-8* mRNA expression levels were measured using real-time PCR. Each transcript level was normalized to corresponding *β-actin* value and is expressed as relative units to untreated control. (**c**) Effects of Cyto D (0.5 μM) on the release and mRNA expression levels of IL-8 in dHL-60 cells exposed to 100 μg/mL _high_Fe-CNT. Values are means ± SD (n = 3). **P < 0.01 (versus untreated control, Dunnett, ANOVA). Statistical comparisons between the two groups were carried out using the Student’s *t*-test.

The above results indicated that the cytotoxicity of MWCNTs toward dHL-60 was predominantly caused by neither the acellular nor intracellular ROS generation. Then, we hypothesized that accumulation of excess iron in the dHL-60 cells leads to induction of inflammatory response. Indeed, the regulation of iron homeostasis is tightly linked to inflammatory response^43^. To cite an example, the reduction of intracellular iron by chelation exerts an inhibitory effect on cytokine production^44,45^. In addition, the intracellular accumulation of free iron, which is caused by iron-based nanoparticle exposure, resulted in the destruction of iron homeostasis and the increase of oxidative stress and inflammatory response^46^. Then, dHL-60 cells were incubated with MWCNTs for 24 h and the amount of IL-8 protein released into the culture medium was measured. The amount of IL-8 protein released from dHL-60 cells increased in a dose-dependent manner after incubation with _High_Fe-CNT, whereas no such effect was observed in udHL-60 cells (Fig. 3a). On the other hand, IL-8 protein expression levels were not increased in _Low_Fe-CNT-treated dHL-60 cells, indicating that the intracellular accumulation of iron caused IL-8 production. In addition, the IL-8 protein release caused by the exposure to _High_Fe-CNT was attenuated by the addition of Cyto D (Fig. 3c, left panel). Consistent with the IL-8 protein expression levels, *IL-8* mRNA expression levels increased after 24 h exposure to _High_Fe-CNT and decreased on treatment with Cyto D (Fig. 3b,c, right panel), whereas no effect was observed in _Low_Fe-CNT-treated cells (Fig. 3a,b). These results indicated that the intracellular accumulation of iron released from MWCNTs in the acidic lysosomal environment induces IL-8 expression in dHL-60 cells.

### Effects of MWCNTs on intracellular calcium concentration ([Ca^2+^]_i_)

**Figure 4:**
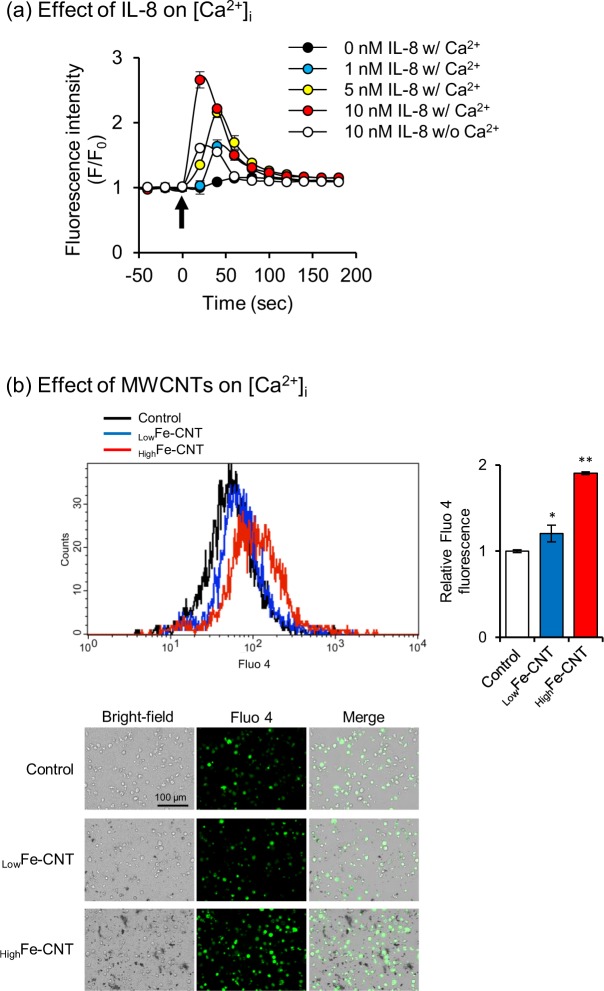
Effect of IL-8 and MWCNTs on intracellular calcium concentration ([Ca^2+^]_i_). (**a**) Effects of IL-8 stimuli on [Ca^2+^]_i_ in dHL-60 cells. Cells were loaded with Fluo 4-AM for 1 h and then stimulated with various concentrations of IL-8 in the presence or absence of extracellular calcium. Arrow indicates time of IL-8 addition. (**b**) Effects of MWCNTs on [Ca^2+^]_i_ in dHL-60 cells. dHL-60 cells were exposed to 100 μg/mL MWCNTs for 24 h and stained with Fluo 4-AM for 1 h. Then, they were analyzed by flow cytometry and visualized under a fluorescence microscope. Values are means ± SD (n = 3). *P < 0.05, **P < 0.01 (versus untreated control, Dunnett, ANOVA).

IL-8 is a chemokine that is produced by many cell types, including monocytes, macrophages, and epithelial and endothelial cells, in response to inflammatory stimuli, and functions to activate and recruit neutrophils. Several studies have revealed that the activation of neutrophils by IL-8 results in the increase of [Ca^2+^]_i_^47–49^. We confirmed that IL-8 triggered [Ca^2+^]_i_ elevation in a concentration-dependent manner, whereas the IL-8-induced [Ca^2+^]_i_ elevation was reduced in the absence of extracellular Ca^2+^ (Fig. 4a), as reported previously^48^. Then, we investigated the effects of MWCNTs on [Ca^2+^]_i_ in dHL-60 cells and found a significant increase in [Ca^2+^]_i_ when dHL-60 cells were exposed to _High_Fe-CNT by both flow cytometry and fluorescence microscopy (Fig. 4b). The [Ca^2+^]_i_ elevation by the treatment with _Low_Fe-CNT was less than that with _High_Fe-CNT. These results suggest that the increase of [Ca^2+^]_i_ caused by _High_Fe-CNT is induced by the accumulation of extracellular IL-8.

**Figure 5:**
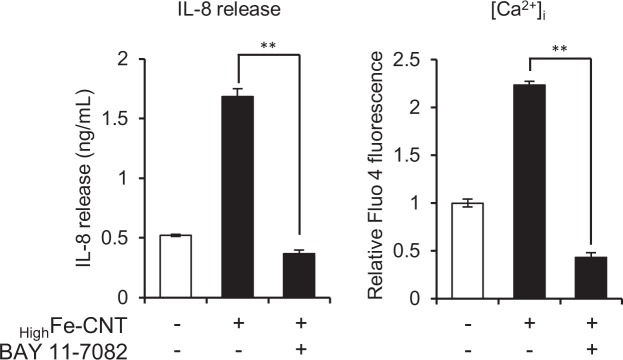
Effect of BAY 11–7082 on IL-8 release and [Ca^2+^]_i_. dHL-60 cells were pretreated with 5 μM BAY 11–7082 for 30 min and then exposed to 100 μg/mL _high_Fe-CNT for 24 h. IL-8 release into culture medium was determined by ELISA. [Ca^2+^]_i_ in dHL-60 cells was determined using Fluo 4-AM and flow cytometry. Values are means ± SD (n = 3). Statistical comparisons between the two groups were carried out using the Student’s *t*-test.

On the contrary, several studies have indicated that IL-8 gene expression is induced by the increase of [Ca^2+^]_i_ in various cell types, including neutrophils, monocytes, and epithelial cells^50–53^. We also reported that [Ca^2+^]_i_ elevation resulted in an increase of IL-8 production in macrophages using calcium-based fine particles^54^. Then, to investigate whether [Ca^2+^]_i_ elevation by _High_Fe-CNT is caused by IL-8 stimulus, dHL-60 cells were pre-treated with BAY 11–7082, an inhibitor of NF-κB activation, for 30 min and subsequently exposed to _High_Fe-CNT for an additional 24 h. Treatment of dHL-60 cells with BAY 11–7082 resulted in the attenuation of the increase of IL-8 production caused by _High_Fe-CNT exposure (Fig. 5, right panel). Interestingly, the increase of [Ca^2+^]_i_ triggered by the exposure to _High_Fe-CNT was also significantly repressed by the pre-treatment with BAY 11–7082, indicating that [Ca^2+^]_i_ elevation in dHL-60 cells requires IL-8 production through NF-κB activation. These results suggest that the intracellular Ca^2+^ homeostasis of dHL-60 cells is perturbed by the exposure to _High_Fe-CNT *via* the iron-dependent production of IL-8.

### Induction of apoptosis by MWCNTs

**Figure 6:**
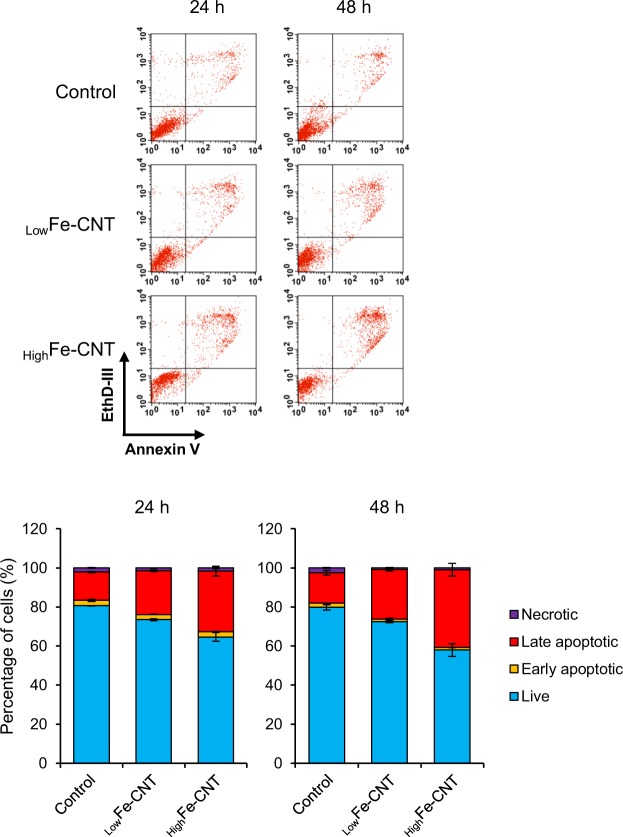
Effect of MWCNTs on apoptosis in dHL-60 cells. (**a**) dHL-60 cells were treated with 100 μg/mL MWCNTs for 24 and 48 h. The cells were stained with FITC-Annexin V and EthD-III for 15 min and then analyzed by flow cytometry. In each panel, the lower left quadrant shows healthy cells (Annexin V−/EthD-III−). Lower right, upper right, and upper left quadrants show early apoptosis (Annexin V+/EthD-III−), late apoptosis (Annexin V+/EthD-III+), and necrosis (Annexin V−/EthD-III+), respectively. (**b**) Data are summarized and expressed as percentage of total cell number. Values are means ± SD (n = 3).

Calcium plays an essential role in the physiology and biochemistry of cells and organisms, and is one of the most commonly used intracellular second messengers. However, it has been reported that sustained [Ca^2+^]_i_ elevation triggers the mitochondria-mediated apoptotic pathway^55^. Then, to investigate whether MWCNTs induce apoptotic cell death, dHL-60 cells were exposed to MWCNTs for 24 and 48 h, and the numbers of healthy, apoptotic, and necrotic cells were measured by Annexin V and EthD-III staining. As shown in Fig. 6, clear increases in the number of apoptotic cells, particularly the late apoptotic cells (Annexin V+/EthD-III+), were observed in MWCNT-exposed cells, and the ratio of apoptotic cells exposed to _High_Fe-CNT was higher than that exposed to _Low_Fe-CNT. On the other hand, the treatment of dHL-60 cells with 100 to 500 μM iron(II) chloride exhibited little effects on the IL-8 production and apoptosis due to the failure of intracellular accumulation of iron (data not shown), indicating the iron overload caused by engulfed MWCNTs triggered apoptotic cell death. In addition, to investigate the involvement of ROS in apoptotic cell death caused by MWCNTs, cells were treated with *N*-acetyl-l-cysteine (NAC) for 1 h before exposure to MWCNTs; subsequently, we measured the number of apoptotic cells after 24 h. As shown in Supplementary Fig. S4, the pretreatment with NAC almost completely inhibited _Low_Fe-CNT-induced apoptotic cell death. However, NAC did not inhibit _high_Fe-CNT-induced apoptotic cell death completely. These results suggest that _high_Fe-CNT-induced apoptotic cell death is additively induced by intracellular accumulation of ROS and ferrous iron.

To confirm the apoptosis of MWCNT-treated dHL-60 cells, DNA strand breakage was visualized and quantified by the alkaline comet assay, which has long been used to visualize DNA strand breakage as an indicator of genotoxic insult. As shown in Supplementary Fig. S5, DNA strand breakage was induced by the treatment with _High_Fe-CNT but not _Low_Fe-CNT. Although it has been widely considered that iron-mediated DNA strand breakage is caused by iron-mediated ROS generation^56^, our results indicate no correlation among the amount of intracellular iron (Fig. 2b), intracellular ROS generation (Supplementary Fig. S3b), and DNA strand breakage (Supplementary Fig. S5). Therefore, _High_Fe-CNT-mediated DNA strand breakage is related to the activation of the apoptotic pathway.

**Figure 7:**
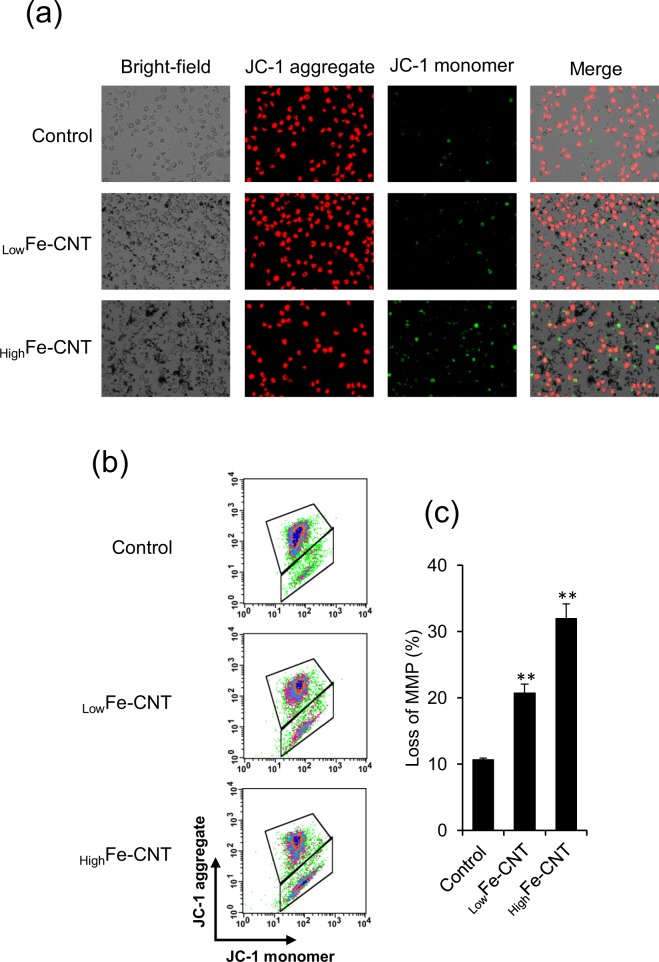
Effect of MWCNTs on mitochondrial membrane potential (MMP) of dHL-60 cells. dHL-60 cells were treated with 100 μg/mL MWCNTs for 24 h. The cells were stained with JC-1 and visualized under a fluorescence microscope (**a**) or analyzed by flow cytometry (**b**). The scatter plots of the flow cytometry analysis show the distribution of JC-1 aggregates and JC-1 monomer. (**c**) The graph shows the percentage of JC-1 monomer-positive cells. Values are means ± SD (n = 3). **P < 0.01 (versus untreated control, Dunnett, ANOVA).

Mitochondria play important roles in apoptotic signals triggered by both extrinsic and intrinsic apoptotic pathways^57,58^. To confirm the possibility that MWCNT-induced apoptosis is related to the mitochondrial signaling pathways, dHL-60 cells were treated with MWCNTs for 24 h and changes in mitochondria membrane potential (MMP) and caspase-3 activities were examined. Changes in MMP induced by MWCNTs were assessed by using JC-1 as the fluorescence probe and analyzed by fluorescence microscopy and flow cytometry. In healthy cells that have high MMP levels, JC-1 emits red fluorescence. In contrast, in apoptotic or unhealthy cells that have low MMP levels, JC-1 emits green fluorescence. Representative fluorescence microscopy images are shown in Fig. 7a; treatment with _High_Fe-CNT caused a red to green color shift, indicating loss of MMP. To perform a quantitative analysis of MMP, we also investigated the change in MMP levels of MWCNT-exposed cells by flow cytometry (Fig. 7b,c). Consistent with the observation by fluorescence microscopy, MWCNTs induced a significant decline in MMP levels in dHL-60 cells. As the loss of MMP is a crucial event in apoptotic cell death^59,60^, our results indicated that _High_Fe-CNT induced apoptosis in dHL-60 cells through the mitochondria-mediated apoptotic pathway.

**Figure 8:**
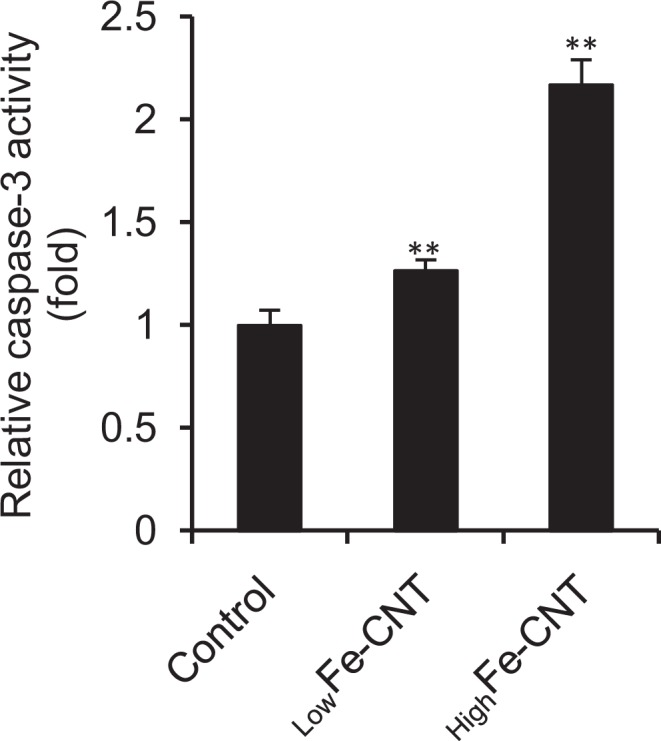
Effect of MWCNTs on caspase-3 activity of dHL-60 cells. dHL-60 cells were treated with 100 μg/mL MWCNTs for 24 h. Caspase-3 activity in total cell lysates was measured using the caspase-3 substrate, Z-DEVD-R110. The activity was normalized to total protein concentration and presented as relative units to untreated control. Values are means ± SD (n = 3). **P < 0.01 (versus untreated control, Dunnett, ANOVA).

Caspases belong to a family of cysteine proteases that mediate the apoptotic pathway^61,62^. Among them, caspase-3 is considered the most crucial protease as it is essential for apoptotic cell death in mammalian cells^63^. As shown in Fig. 8, a clear increase of caspase-3 activity was observed in _High_Fe-CNT-exposed dHL-60 cells. Although slight decrease of MMP and increase of caspase-3 activity also observed in _low_Fe-CNT-exposed cells (Figs 7 and 8), our results suggest that _low_Fe-CNT-induced cytotoxicity is mainly mediated by the intracellular ROS generation (Supplementary Fig. S4). Taken together, these results suggest that calcium homeostasis imbalance and mitochondrial damage are involved in MWCNT-induced apoptosis.

## Conclusion

In this study, we investigated the effects of two types of MWCNTs containing high or low amounts of iron on human promyelocytic leukemia HL-60 cells. We found that the MWCNTs were cytotoxic toward neutrophil-like differentiated HL-60 cells but not undifferentiated HL-60 cells, owing to their high phagocytic activity. In addition, our results indicated that iron was released from engulfed MWCNTs in the acidic lysosomal environment, accumulated in the cells where they accelerated the production of IL-8, and activated the mitochondria-mediated apoptotic pathway. However, MWCNTs that released low amounts of iron did not significantly induce IL-8 production or apoptosis. Together, these results should pave the way for the development of guidelines for carbon nanomaterial production, such as high-efficiency purification procedures, which would enable us to minimize MWCNT-associated health risks and avoid unnecessary prohibition of beneficial MWCNTs.

## Materials and Methods

### HL-60 cell culture and differentiation

Human promyelocytic leukemia HL-60 cells (RCB3683) were obtained from the RIKEN BioResource Center (Ibaraki, Japan) and cultured in RPMI 1640 (Gibco Life Technologies, Gaithersburg, MD, USA) containing 10% heat-inactivated fetal bovine serum (FBS; HyClone Laboratories, Inc., South Logan, UT, USA), 100 U/mL penicillin, 100 μg/mL streptomycin, and 250 ng/mL amphotericin B (Nacalai Tesque Inc., Kyoto, Japan) at 37 °C in an atmosphere of 5% CO_2_. To induce differentiation, 2 × 10^5^ cells/mL were resuspended in complete RPMI 1640 growth medium containing 1.3% DMSO and incubated for 1–4 days. Differentiation of HL-60 cells into a neutrophil-like state was assessed by measuring the levels of CD11b on the cell surface. For the detection of CD11b, cells were harvested, washed with phosphate-buffered saline (PBS), and stained for 30 min with APC-conjugated anti-human CD11b antibody on ice in the dark. The stained samples were analyzed using a FACSCalibur flow cytometer (Becton, Dickinson and Company, Franklin Lakes, NJ, USA) (Supplementary Fig. S6).

### Preparation of MWCNT-medium dispersions

Sample-A and Sample-B were produced by Showa Denko K.K. (Tokyo, Japan) and Hodogaya Chemical Co., Ltd. (Tokyo, Japan), respectively. The MWCNTs were dispersed in 0.5% Pluronic F-68 (Sigma-Aldrich, St. Louis, MO, USA) at the concentration of 1 mg/mL. Stock solutions were sonicated for 30 min before being diluted in culture medium and applied to HL-60 cells. Hydrodynamic diameter of the MWCNTs in the culture medium was determined with a dynamic light scattering nanoparticle size analyzer (LB-550; HORIBA Ltd, Kyoto, Japan) at 25.0 °C ± 0.1 °C. The light source was a 650-nm laser diode of 5 mW.

### Measurement of iron released from MWCNTs

The evaluation of iron released from MWCNTs was performed in culture medium, in acidic condition (20 mM citrate buffer, pH 4.5) and neutral condition (20 mM sodium phosphate buffer, pH7.2). MWCNTs were dispersed in culture medium, citrate buffer or sodium phosphate buffer at the concentration of 100 μg/mL by sonication, and then incubated at 37 °C for 24 h. The MWCNTs were separated from the solution by ultrafiltration using a Microcon Ultracel YM-3 (MWCO: 3000, Millipore Corporation, Bellerica, MA, USA). The filtrate was collected and the amount of iron was measured with an ICP-MS (X-Series II; ThermoFisher Scientific Inc., Waltham, MA, USA).

### Cell viability assay

Cell viability was measured by the WST-1 assay (Takara Bio, Shiga, Japan) according to the manufacturer’s instructions. To determine whether MWCNTs interfere with the WST-1 reaction product, WST-1 interference test was performed. The healthy cells were incubated with WST-1 solution for 1 h at 37 °C in 5% CO_2_, and then mixed with 100 μg/mL MWCNTs for 1 h. Subsequently, the reaction mixture was centrifuged at 10000 × *g* for 10 min and measured at 450 nm using a Tecan Infinite M200 (Tecan Group Ltd.). The results indicated that the interference of the MWCNTs with WST-1 assay was negligible (data not shown).

### Determination of intracellular iron level

Intracellular iron levels were measured according to a previously described method^64^ with some modifications. dHL-60 cells exposed to MWCNTs were rinsed with culture medium three times and resuspended in PBS. The cells were incubated with 5 μM RhoNox-1 (Goryo Chemical, Inc., Sapporo, Japan) in the dark at 37 °C for 1 h and then washed with PBS three times. Cell analysis was carried out using a FACSCalibur flow cytometer (Becton, Dickinson and Company). RhoNox-1 was excited at 488 nm and measured at 575 nm. For the visualization of intracellular iron, cells stained with RhoNox-1 were observed under a BZ-X710 all-in-one fluorescence microscope (Keyence, Osaka, Japan).

### Determination of acellular and intracellular ROS generation

To measure acellular ROS generation, 2.5 mL of methanol and 10 mL of 10 mM NaOH were added to 0.5 mL of 5 mM DCFH-DA (Sigma-Aldrich). The reaction was carried out for 30 min at room temperature in the dark and stopped by adding PBS. Then, final concentration of 5 μM DCFH and 100 μg/mL MWCNTs were mixed in culture medium and DCF fluorescence was measured for 5 h using a Tecan Infinite M200 (Tecan Group Ltd., Männedorf, Switzerland) at the excitation wavelength of 485 nm and the emission wavelength of 530 nm. To measure intracellular ROS generation, cells were incubated with MWCNTs and subsequently rinsed with culture medium three times. After 30 min incubation with 10 μM DCFH-DA in serum-free RPMI 1640, the cells were analyzed using a FACSCalibur flow cytometer (Becton, Dickinson and Company) at the excitation wavelength of 488 nm and the emission wavelength of 525 nm.

### IL-8 enzyme-linked immunosorbent assay (ELISA)

Cells were cultured with MWCNTs for 24 h. IL-8 protein levels in the cell supernatants were measured using an ELISA Kit (eBioscience, San Diego, CA, USA) following the manufacturer’s instructions.

### Isolation of total RNA and quantitative real-time PCR

Total RNA was extracted using an RNeasy Mini Kit (Qiagen, Valencia, CA, USA) according to the manufacturer’s instructions. First-strand cDNA was synthesized using a High Capacity cDNA Reverse Transcription Kit (Applied Biosystems, Foster City, CA, USA) according to the manufacturer’s instructions. *IL-8* mRNA levels were analyzed using the TaqMan Gene Expression Assay (ID: Hs00174103_m1, Applied Biosystems). The housekeeping gene β-actin (ID: Hs99999903_m1) was used as the internal control. Target mRNA levels were measured using an Applied Biosystems 7300 Real-Time PCR System. The *IL-8* expression levels in each sample were normalized to *β-actin* and then compared with untreated controls. The results are reported as fold change relative to control.

### Measurement of intracellular calcium concentration ([Ca^2+^]_i_)

To investigate the effect of IL-8 on [Ca^2+^]_i_, changes of [Ca^2+^]_i_ were measured by using a previously described method^48^ with some modifications. dHL-60 cells were loaded with 5 μg/mL Fluo 4-AM (Dojindo Laboratories, Kumamoto, Japan) in physiological salt solution (PSS) [115 mM NaCl, 5 mM KCl, 1 mM KH_2_PO_4_, 10 mM glucose, 1 mM MgSO_4_, 1.25 mM CaCl_2_, 25 mM HEPES (pH 7.4), and 0.1% bovine serum albumin (BSA)] at 37 °C for 1 h. Thereafter, the cells were washed with PSS three times and resuspended in PSS with or without CaCl_2_. The Fluo 4-AM-loaded cells were maintained at 37 °C for 5 min in Tecan Infinite M200 (Tecan Group Ltd.) and then recombinant IL-8 (R&D Systems, Minneapolis, MN, USA) was added to the medium. Fluo 4 fluorescence was measured at the excitation wavelength of 490 nm and the emission wavelength of 520 nm and recorded in real time at 20 sec intervals. The results are presented as background-subtracted normalized fluorescence (F/F_0_) where F is fluorescence intensity and F_0_ is average fluorescence obtained before the addition of IL-8.

To investigate the effect of MWCNTs on [Ca^2+^]_i_ in dHL-60 cells, the cells were incubated with MWCNTs for 24 h. After washing the cells with culture medium and PBS, the cells were loaded with 5 μg/mL Fluo 4-AM and 0.02% Pluronic F-127 at 37 °C for 1 h. Thereafter, the cells were analyzed with a FACSCalibur flow cytometer (Becton, Dickinson and Company) at the excitation wavelength of 488 nm and the emission wavelength of 525 nm. For visualization of Fluo 4 fluorescence, the cells were observed under a BZ-X710 all-in-one fluorescence microscope (Keyence).

### Apoptosis assay

Cell death induced by MWCNTs was assessed using an Apoptotic/Necrotic Cells Detection Kit (Promokine, Heidelberg, Germany) according to the manufacturer’s instructions. In brief, dHL-60 cells exposed to MWCNTs were rinsed with culture medium three times and resuspended in PBS. After that, the cells were stained with FITC-Annexin V and Ethidium Homodimer III (EthD-III) for 15 min in the dark at room temperature. Cellular status was analyzed using a FACSCalibur flow cytometer (Becton, Dickinson and Company). Cellular status was determined as follows: early apoptosis (Annexin V+/EthD-III−), late apoptosis (Annexin V+/EthD-III+), necrosis (Annexin V−/EthD-III+), and healthy cells (Annexin V−/EthD-III−). To determine whether MWCNTs interfere with the apoptosis assay, the interference test was performed. The cells were incubated with 1 μM staurosporine (STS; Wako Pure Chemical Industries, Osaka, Japan) for 24 h at 37 °C in 5% CO_2_. After that, the cells were stained with FITC-Annexin V and EthD-III as described above. Subsequently, the stained cells were mixed with 100 μg/mL MWCNTs prior to the flow cytometric analysis. The results indicated that the interference of the MWCNTs with apoptosis assay was negligible (data not shown).

### Alkaline comet assay

The alkaline comet assay was performed according to the manufacturer’s instructions (Comet Assay, Trevigen, Gaithersburg, MD, USA). In brief, dHL-60 cells exposed to MWCNTs were rinsed with culture medium three times and resuspended in PBS. The cell suspension was mixed with LMAgarose and immediately transferred onto CometSlides. After cell lysis at 4 °C, the CometSlides were treated with an alkaline unwinding solution (0.2 M NaOH and 1 mM EDTA, pH > 13) for 60 min. Electrophoresis was performed at 20 V for 30 min at 4 °C in the dark and the separated products were stained with a silver-staining kit (Trevigen). Comet tail length was defined as the distance between the leading edge of the nucleus and the end of the tail. Data were collected from 150 cells per experiment in triplicate (50 cells/slide/culture).

### JC-1 staining

Mitochondrial membrane potential (MMP) was determined with a JC-1 Mitochondrial Membrane Potential Assay Kit (Cayman Chemical Company, MI, USA) according to the manufacturer’s instructions. In brief, dHL-60 cells exposed to MWCNTs were rinsed with culture medium three times and resuspended in PBS. After that, the cells were stained with JC-1 working solution for 30 min and then analyzed using a FACSCalibur flow cytometer (Becton, Dickinson and Company). The emission wavelengths of JC-1 monomer and JC-1 aggregates were 530 and 590 nm, respectively. For visualization of JC-1 fluorescence, the cells were observed under a BZ-X710 all-in-one fluorescence microscope (Keyence).

### Measurement of caspase-3 activity

Caspase-3 activity was measured with an EnzChek Caspase-3 Assay Kit #2, Z-DEVD-R110 substrate (ThermoFisher Scientific Inc.) according to the manufacturer’s instructions. In brief, dHL-60 cells exposed to MWCNTs were lysed with 50 μL of cell lysis buffer and centrifuged. The supernatants were mixed with Z-DEVD-R110 substrate at room temperature for 30 min in the dark. Thereafter, the fluorescence intensities were measured using a Tecan Infinite M200 (Tecan Group Ltd.) at the excitation wavelength of 495 nm and the emission wavelength of 520 nm. To determine whether MWCNTs interfere with the caspase-3 assay, rhodamine 110 (R110) interference test was performed. The cells were incubated with 1 μM STS (Wako Pure Chemical Industries) for 24 h at 37 °C in 5% CO_2_. Subsequently, the cells were lysed and mixed with Z-DEVD-R110 substrate as described above. Before measurement of fluorescence intensities, the MWCNTs were mixed with reaction product at the final concentration of 100 μg/mL for 1 h. The mixture was centrifuged at 10000 × *g* for 10 min and measured at the excitation wavelength of 495 nm and the emission wavelength of 520 nm. The results indicated that the interference of the MWCNTs with R110 was negligible (data not shown).

### Statistical analysis

All assays were conducted in triplicate at least. Data are expressed as means and standard deviation (SD). Statistical analyses were performed by analysis of variance (ANOVA) using the Dunnett test for multiple comparisons. The Student’s *t*-test was used to determine the statistical significance of differences between two groups.

## Supplementary information

Supplementary Figures
