# Supplementary material for: Effect of iron overload from multi walled carbon nanotubes on neutrophil-like differentiated HL-60 cells
Source: Sci Rep. 2019 Feb 18;9:2224. doi: 10.1038/s41598-019-38598-4 (PMC6379482; doi:10.1038/s41598-019-38598-4)
Supplement: Supplementary file 1 — Supplementary Figures [file 41598_2019_38598_MOESM1_ESM.pdf]

Effect of iron overload from multi walled carbon nanotubes on neutrophil-like differentiated HL-60 cells

Yosuke Tabei[1,*], Hiroko Fukui[2], Ayako Nishioka[2], Yuji Hagiwara[2], Kei Sato[2], Tadashi Yoneda[2], Tamami Koyama[3], Masanori Horie[1]

[1]Health Research Institute, National Institute of Advanced Industrial Science and Technology (AIST), 2217-14 Hayashi-cho, Takamatsu, Kagawa 761-0395, Japan
[2]Safety Evaluation Center, Showa Denko K.K., 1-1-1 Ohnodai, Midori-ku, Chiba-shi, Chiba 267-0056, Japan
[3]Institute for Advanced and Core Technology, Showa Denko K.K., 1-1-1 Ohnodai, Midori-ku, Chiba-shi, Chiba 267-0056, Japan

[*]Corresponding author
Address: Health Research Institute, National Institute of Advanced Industrial Science and Technology (AIST), 2217-14 Hayashi-cho, Takamatsu, Kagawa 761-0395, Japan.
E-mail: y-tabei@aist.go.jp.
Tel: +81-87-869-3598
Fax: +81-87-869-3553

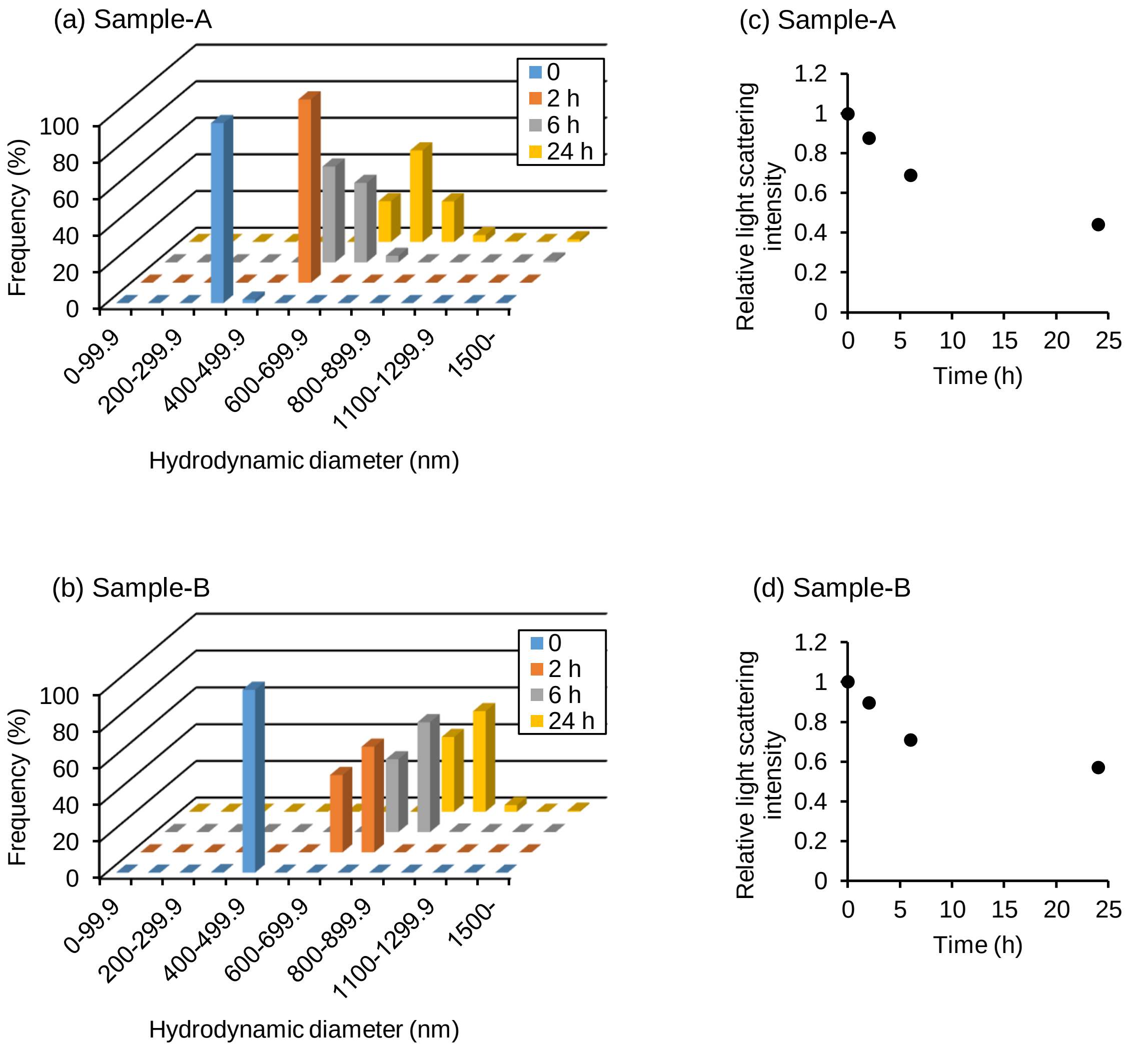

Fig. S1. Characteristics of MWCNT medium dispersions used in this study.
(a and b) Histograms of hydrodynamic diameter of MWCNTs based on particle number measured by DLS. (c and d) Stabilities of MWCNTs in the medium dispersions. Light scattering intensities of MWCNT medium dispersions were measured by DLS.

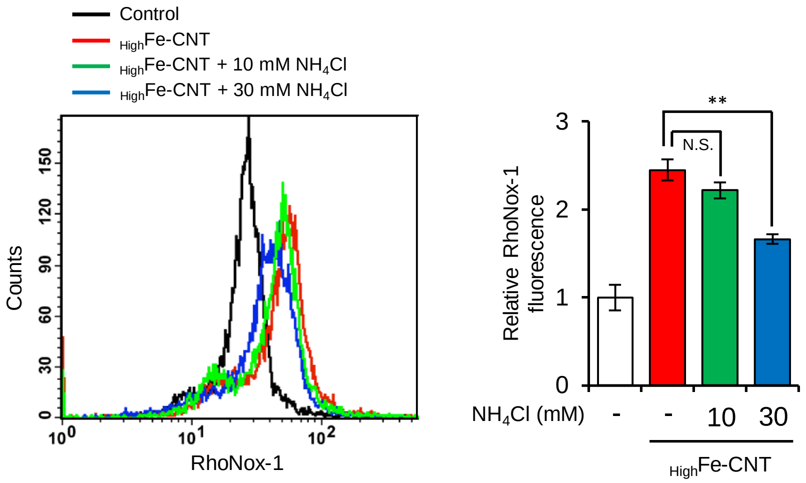

Fig. S2. Effect of ammonium chloride on intracellular accumulation of iron. dHL-60 cells were treated with 10 or 30 mM ammonium chloride and 100 μg/mL $_{High}$Fe-CNT for 24 h, and stained with RhoNox-1 for 1 h. Then, they were analyzed by flow cytometry. Values are means ± SD (n = 3). Statistical comparisons between the two groups were carried out using the Student's *t*-test.

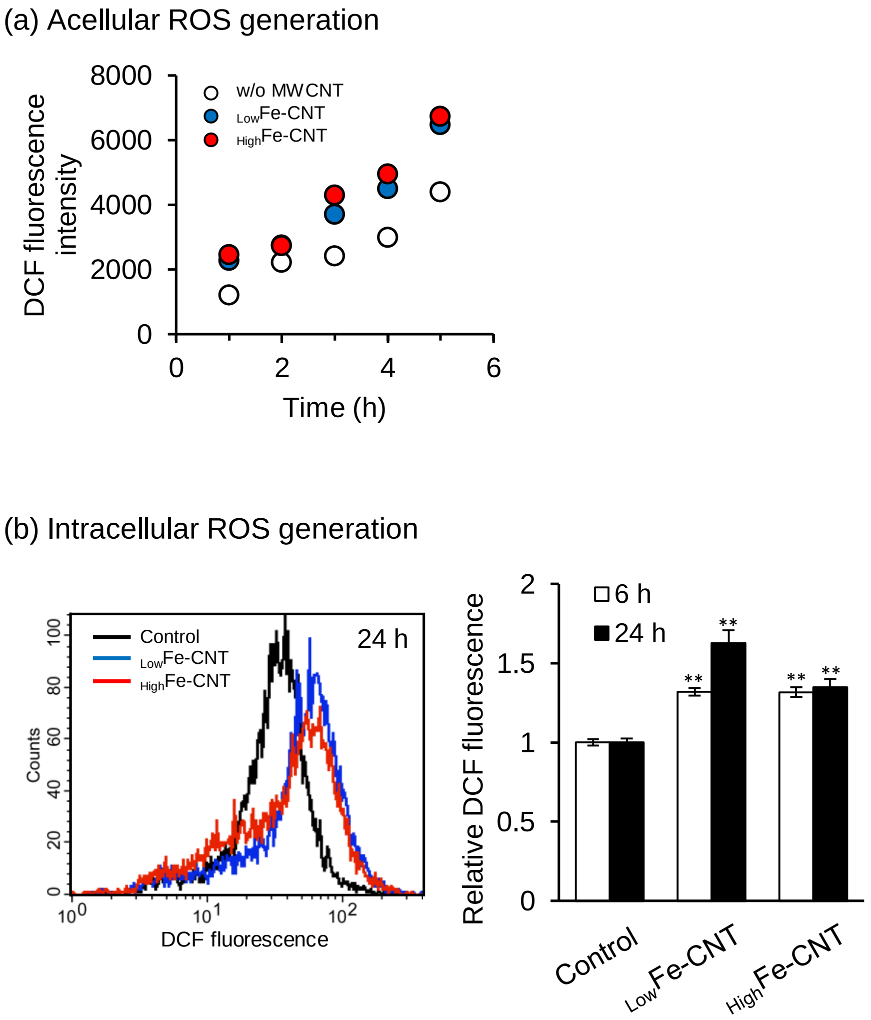

Fig. S3. Acellular and intracellular ROS generation. (a) Acellular ROS generation after addition of MWCNTs to DCFH solution. Fluorescence was measured at the excitation wavelength of 485 nm and the emission wavelength of 530 nm. Measurements were performed at 1, 2, 3, 4, and 5 h after the addition of MWCNTs. (b) Intracellular ROS levels in dHL-60 cells treated with MWCNTs. dHL-60 cells were treated with 100 μg/mL MWCNTs for 6 and 24 h, and intracellular ROS levels were measured by the DCFH method using flow cytometry. Results are presented as relative units to untreated control. Values are means ± SD (n = 3). ** $P < 0.01$ (versus untreated control, Dunnett, ANOVA).

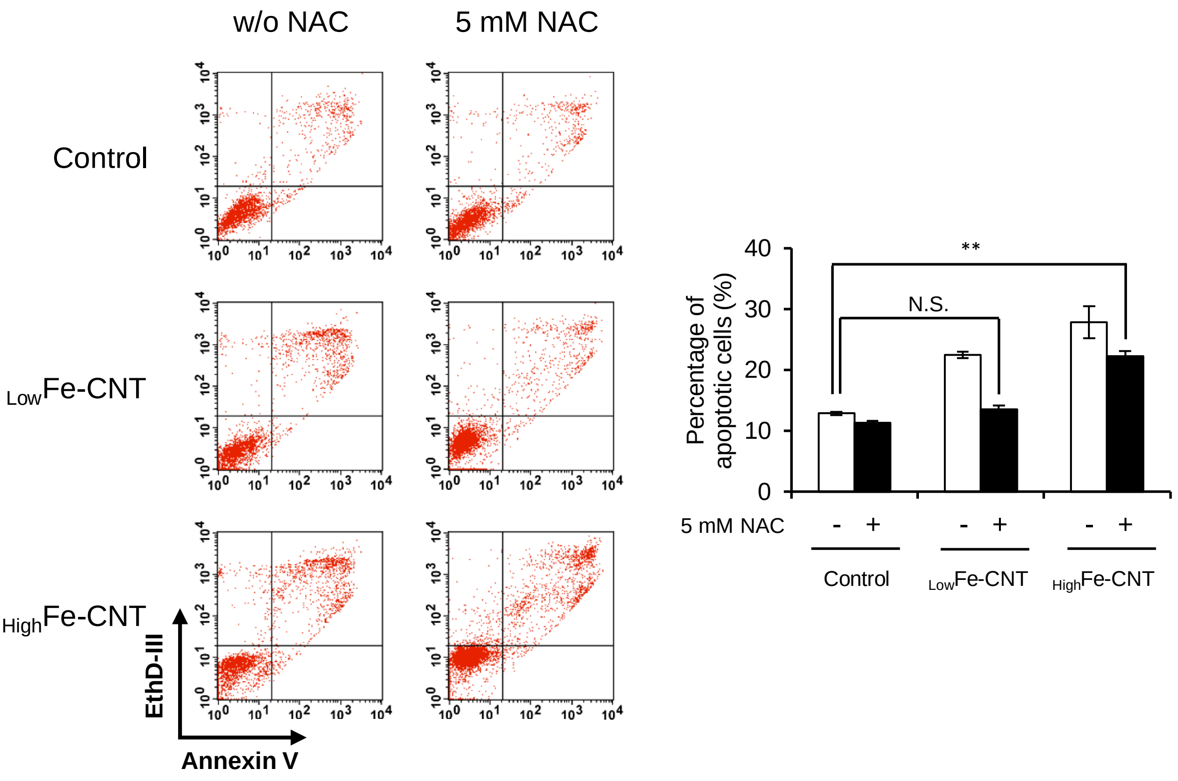

Fig. S4. Effect of NAC on MWCNT-induced apoptosis. dHL-60 cells were pretreated with 5 mM NAC for 1 h and then exposed to 100 μg/mL MWCNTs for 24 h. After that, cells were stained with FITC-Annexin V and EthD-III for 15 min and then analyzed by flow cytometry. Cells positive for Annexin V staining were considered apoptotic. Values are means ± SD (n = 3). Statistical comparisons between the two groups were carried out using the Student's *t*-test.

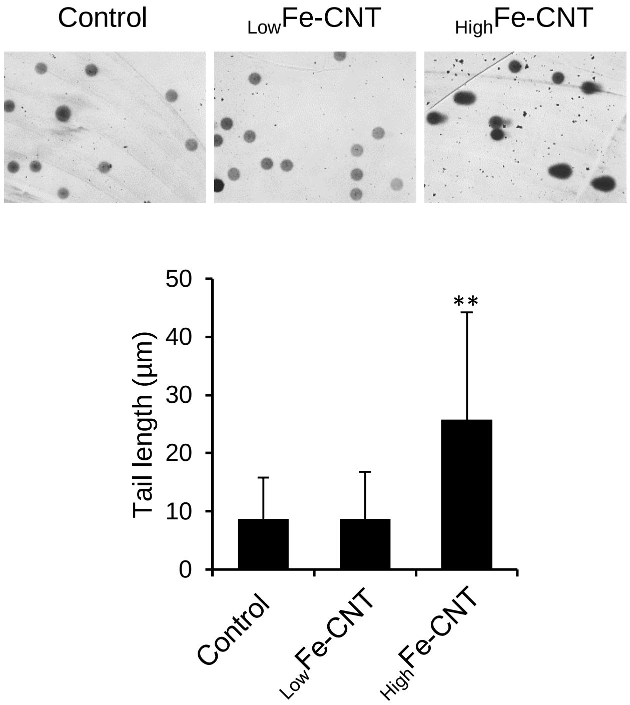

Fig. S5. Effect of MWCNTs on DNA integrity. dHL-60 cells were exposed to 100 µg/mL MWCNTs for 24 h and then DNA strand breakage was assessed by the alkaline comet assay. Tail length values of DNA were obtained by analyzing at least 50 random comet images from each treatment. Upper panels are comet images of untreated control and MWCNT-treated cells at 24 h. ** $P < 0.01$ (versus untreated control, Dunnett, ANOVA).

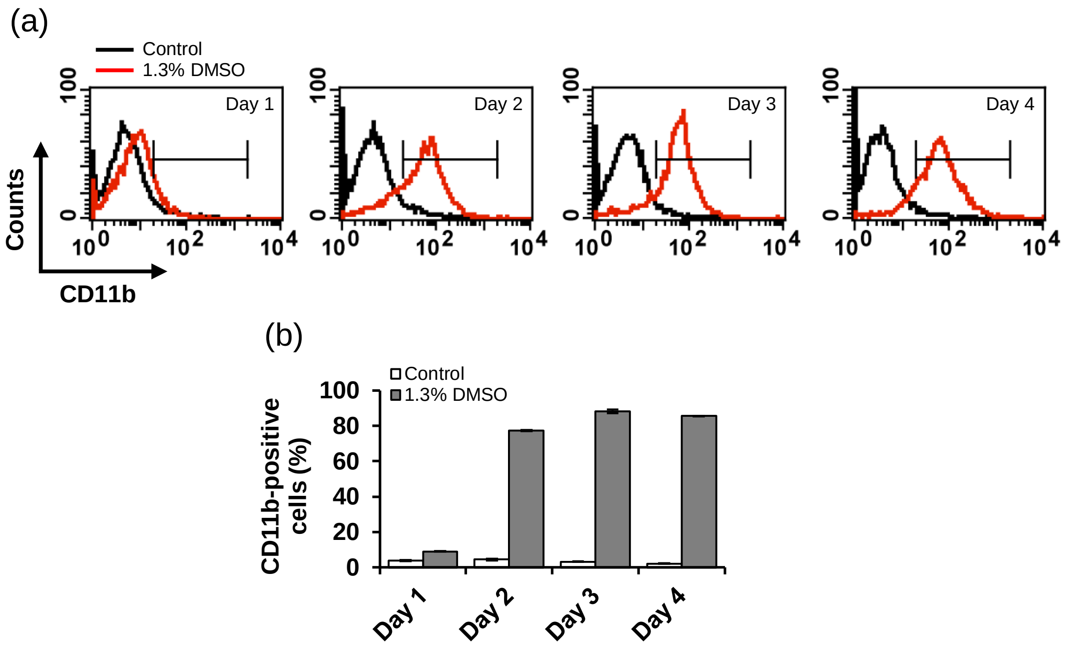

Fig. S6. Flow cytometric analysis of CD11b expression in HL-60 cells upon treatment with 1.3% DMSO. (a) HL-60 cells were incubated for 1 to 4 days in the presence of 1.3% DMSO and stained with APC-conjugated anti-human CD11b antibody. Stained samples were analyzed using a flow cytometer. (b) The graph shows the percentage of CD11b-positive cells. Values are means ± SD (n = 3).
